# CaseConferencing: telecom resource used for an original approach to on-going teaching through case expertise

**DOI:** 10.1186/1746-1596-8-S1-S35

**Published:** 2013-09-30

**Authors:** Jacques Klossa, Frédérique Capron, Dominique Algalarrondo, Gilles Le Nahour, Jean-François Pomerol

**Affiliations:** 1 TRIBVN, France; 2 CHU La Pitié, France; 3 Orange, France

## Introduction

In the pathology field, digital slide conferencing capabilities are obviously a highly necessary functionality when multiple participants need to simultaneously view the same digital slide from multiple, remote locations e.g. for frozen section teleconsultation, second opinion on complex cases or e-learning. In this presentation we present an expertise approach that uses teleconsultation to transfer the knowledge from the expert to requesters. In that context, we will also analyze advantages and perspectives in integrating a general purpose solution to a business specialty (pathology) workflow.

## Methods

The idea from the department of Pathology in La Pitié University Hospital center is to take advantage from whole slide image (WSI) teleconsultation to provide an on-going teaching service that will enhance the interpretation level of unspecialized pathologists. Slides are sent to La Pitié for expertise; they are immediately scanned and sent back the same day to the emitter service for storage.

For such application the Orange Multimedia Conference module which is a general purpose piece of software has been integrated to the TRIBVN Calopix™ platform. Calopix™ platform is a dedicated pathology platform which is integrated in the hospital workflow between LIS (Laboratory Information System) and PACS. Multimedia Conference is a web conference service widely used and many thousands conferences are opened daily. To meet the healthcare needs the video module has been enhanced for high image quality, large screen compatibility and exchanges traceability. Such collaboration brings secure data transmission and image compression knowhow to pathology business.

## Results

The resulting CaseConferencing module allows recently scanned WSI, i.e. for frozen sections, to be shared instantly without any previous server upload between the conference organizer (expert) and up to 25 participants (case emitters and other interested pathologists). The organizer uses the pathology image workstation that allows classical pan and zoom functions as well as annotation or image analysis tools. Invited participants are informed by email and may participate through the use any web browser on PC or Mac. Among main functionalities, session leadership can be transferred to any participant and real time annotations are automatically stored on the organizer PC.

CaseConferencing guarantees higher quality images transmission and speed for a known transmission channel in addition to ensure security and annotation traceability. Such high quality transmitted image allows clear visualization of very thin features like one pixel thick overlays showing image analysis results. Visualization latency is highly dependent on communication network configuration. It has been generally considered as good enough for WSI sharing and in most cases latency was clearly beyond one second. Such service can be efficiently used as a complement of cooperative asynchronous applications commonly used in the field of telepathology when WSI can be shared through a remote server.

The service is currently used since 2011 by the pathology lab in La Pitié University Hospital in Paris to deliver an expertise service toward private and general hospitals. The purpose is to define guideline describing such expertise services so that it could be extended to other university hospitals.

## Discussion

Visualisation and diagnostic clearly allows on screen diagnostic follow up. However higher "fluidity" of pan and scan would be required to achieve the same feeling as when viewing a slide through a microscope. Another demand that will be answered is the publishing of the session report which has been stored on the organizer PC.

The main purpose of this communication was to evaluate how a platform dedicated to pathology could take profit from existing multipurpose information and telecom tools. Such integration proved to be good enough for case conferencing application. The next step would be to apply such concepts to meet another telepathology issue which needs to share expert knowledge. More specifically, in the Cloud Computing context, the idea would be to use the PaaS (Platform as a service) layer to take profit from the stored patient data information in conjunction with consolidated formalized specialist knowledge to drive WSI exploration and to produce automated pre-annotation that will make easier and quicker their on-line consultation.

## Competing interests

The authors declare that they have no competing interests.

## Authors’ contributions

• FC and GLN provided the original ideas and carried out the evaluation

• JFP in conjunction with DA drove the implementation of the CaseConferencing application to telepathology

• JK and DA contributed to the technical implementation and extended the "CaseConferencing" to "FlexMIm" cloud implementation

